# Assessment and repeatability of aerobic capacity using the Chester Step Test among current, former, and never smokers

**DOI:** 10.1007/s11739-024-03794-2

**Published:** 2024-11-02

**Authors:** Grazia Caci, Lucia Spicuzza, Rosalia Emma, Davide Campagna, Meera Nadir, Erika Anastasi, Francesco Pennisi, Stanley Hunter, Shivraj Bhide, Riccardo Polosa

**Affiliations:** 1 UOC MCAU, University Teaching Hospital “Policlinico-S.Marco”, University of Catania, Via S. Sofia, 78 - Ed. 4, P. 2, 95123 Catania, Italy; 2 Center of Excellence for the Acceleration of HArm Reduction (CoEHAR), University of Catania, Catania, Italy; 3 Department of Clinical and Experimental Medicine, University of Catania, Catania, Italy; 4 Respiratory Unit, University Teaching Hospital “Policlinico-S.Marco”, University of Catania, Catania, Italy; 5 UOC MCAU, University Teaching Hospital “Policlinico-S.Marco”, University of Catania, Catania, Italy; 6 Ashford and St.Peters Hospital, NHS Trust, Chertsey, UK; 7 ECLAT Srl, Spin-off of the University of Catania, Catania, Italy; 8 School of Biosciences, University of Surrey, Guildford, UK; 9 Centre for the Prevention and Treatment of Tobacco Addiction (CPCT), University Teaching Hospital “Policlinico-S.Marco”, University of Catania, Catania, Italy; 10 Department of Medicine and Surgery, ”Kore” University of Enna, Enna, Italy

**Keywords:** Smokers, Aerobic capacity, *V̇*O_2max_, Chester Step Test, Exercise capacity

## Abstract

Cigarette smoking contributes to reduced cardiorespiratory performance, which may improve upon cessation. Consequently, former smokers’ cardiorespiratory fitness should not be significantly different from that of never-smokers. This study aims to compare *V̇*O_2max_ values among current, former, and never smokers and assess the repeatability of measurements using the Chester Step Test (CST). *V̇*O_2max_ measurements were available from a total of 70 subjects (23 current, 23 former, and 24 never-smokers) and showed significant repeatability. Current smokers had the worst aerobic capacity, with a mean *V*O_2max_ ± SD of 38.8 ± 4.5, which was significantly lower than the *V*O_2max_ of 41.62 ± 3.8 in never-smokers (*p* < 0.0001) and 41.43 ± 4.6 in former smokers (*p* < 0.0001). No significant differences were observed between never-smokers and former smokers. *V̇*O_2max_ estimates by CST were reproducible and showed that the aerobic capacity of individuals who smoke is substantially inferior compared to never and former smokers. Improvement in cardiorespiratory performance following smoking cessation may have important implications for smoking cessation, especially for those smokers who perceive enhanced physical performance as a tangible benefit.

## Introduction

V̇O_2max_, the maximum rate of oxygen consumption measured during incremental exercise, is the gold standard for assessing aerobic capacity and indicates improved cardiorespiratory fitness when increased [1–3]. Clinically, low *V̇*O_2max_ is linked to suboptimal cardiorespiratory performance and a higher risk of all-cause mortality [4, 5].

An accurate direct measurement of *V̇*O_2max_ is obtained from a graded aerobic exercise test (GXT), which requires exercise to volitional exhaustion and analysis of the individual’s expired air [6]. This protocol is time-consuming, expensive, ecologically invalid in real-world settings, and may induce high physical stress. Therefore, submaximal exercise testing is commonly used to predict *V̇*O_2max_ when time is limited, laboratory equipment is unavailable, or exercising at high intensities is considered unsafe [7, 8]. Compared to protocols using treadmills, shuttle walks, or cycle ergometers, step tests require only limited equipment (step, heart rate monitor, perceived exertion scale), are safe, inexpensive, simple, portable, and an ecologically valid indirect method of estimating *V̇*O_2max_ [7–9].

There are many step-test protocols, which differ in stepping frequency, test duration, and the number of test stages [10]. These different protocols present differences in the accuracy of *V̇*O_2max_ prediction. For example, it has been suggested that protocols utilizing a fixed step rate may produce a less accurate estimation of cardiorespiratory fitness since a higher exercise intensity is produced in individuals with higher body mass index, lower body height and lower exercise capacity [11].The Chester Step Test (CST) was originally developed by Kevin Sykes at University College Chester to provide a safe and practical means of assessing aerobic capacity by estimating *V̇*O_2max_ [12]. The validity of the CST has been confirmed in healthy subjects for its ability to predict *V̇*O_2max_ compared with a *V̇*O_2max_ measured during a treadmill test, with reasonable error margins ranging from 5 to 15% [13, 14]. For our study we have chosen CST as this test provides the best correlation with measured *V̇*O_2max_ (*r* = 0.95), shows an high test–retest reliability in healthy individuals and the strongest support by validation studies in terms of data collection methods, selection bias, withdrawals and dropouts [10].

Chronic exposure to the harmful chemicals in tobacco smoke is known to severely impair physical fitness. In healthy individuals, smoking can diminish the capacity for both aerobic and anaerobic exercise during physical fitness tests [15–17], primarily due to reduced oxygen uptake capacity at the tissue level [18, 19]. The extent of smoke-related reduced maximal aerobic capacity (*V̇*O_2max_) has been shown to depend on the intensity and duration of exposure to cigarette smoke (i.e., pack-years) [15]. This relationship highlights how smoking impairs the efficiency of the respiratory, cardiovascular, and metabolic systems in oxygen transportation and utilization during exercise. Importantly, the negative effects of smoking on cardiorespiratory performance can be reversed upon cessation [20–22].

The aim of this study is to investigate whether the smoking-induced reduction in aerobic exercise capacity is reversible by comparing *V̇*O_2max_ values among current, former, and never smokers using the Chester Step Test (CST). If smoking’s impact on aerobic capacity is reversible, the *V̇*O_2max_ values of former smokers should closely resemble those of never smokers. To the best of our knowledge, no prior studies have reported changes in aerobic capacity using CST in individuals who have quit smoking. Additionally, there is limited information on the repeatability of *V̇*O_2max_ measurements in current and former smokers. Establishing the repeatability of these measurements is crucial to strengthen the reliability of CST for future clinical research. Therefore, the objectives of this study are: (a) to compare *V̇*O_2max_ values obtained using the CST method between current, former, and never smokers, and (b) to assess the repeatability of these measurements within each group.

## Methods

### Study population

Adult (age 18–50 yrs) current, former, and never smokers were recruited from a pool of people who attended a smoking cessation clinic (CPCT, Centro per la Prevenzione e Cura del Tabagismo of the University of Catania) in the previous 2 years, from hospital staff, and university students.

Current smokers were defined as smokers of ≥ 10 cigarettes per day with an exhaled carbon monoxide (eCO) level of ≥ 7 ppm.

Former smokers were defined as quitters of at least 12 months and who were still abstinent when contacted for enrollment, with an eCO level of < 7 ppm.

Never smokers were defined as having never smoked or who reported having smoked less than 100 cigarettes in their lifetime [23]. Their eCO had to be < 7 ppm to exclude subjects passively exposed to cigarette smoke or environmental sources of carbon monoxide.

Current, former, and never smokers met the following exclusion criteria: (1) history of mental illness, (2) history of alcoholism or drug abuse, (3) presence of any medical conditions or any potential issues that, in the opinion of the investigator, would jeopardize the safety of the participant or preclude completion of the step test, (4) significant exposure to passive smoking (excluding current smokers), (5) use of e-cigarettes or heated tobacco products within the last 3 months, and (6) pregnancy.

The study was approved by the local Ethical Review Board (no. 071/2020/PO, Comitato Etico Catania 1. AOU “Policlinico–V. Emanuele”, Università di Catania, Italy). All participants gave written informed consent before joining the study.

### Study design

This observational study aims to compare *V̇*O_2max_ values obtained using the CST method among three study populations (current, former, and never smokers) and to assess their repeatability. The study consists of a total of three visits: a screening visit, the first visit on day 0 (Visit 1), and a return visit on day 10 (± 2 days) (Visit 2). Subjects were asked to:

- Avoid vigorous exercise/sports before and between sessions
- Avoid heavy meals and alcoholic beverages before study visits
- Not smoke for at least 2 h before each study visit (current smokers only)
- Rest for at least 15 min before starting the step test
- Wear appropriate clothing and footwear during the step test

During the screening visit, subjects received information about the rationale and objectives of the research. Investigators recorded general socio-demographic characteristics (i.e., sex, age, and occupation), verified eligibility criteria, and evaluated smoking status. Subjects were thoroughly reviewed to identify any potential issues that could limit their performance on a step test. All eligible subjects were then invited to attend Visit 1 to review the participant information sheet and sign a consent form. After re-checking inclusion/exclusion criteria, eCO was measured, CST was carried out, and *V*O_2max_ measurements were recorded. Subjects were instructed not to alter their daily exercise patterns and were invited to attend Visit 2. Visit 2 was conducted 10 (± 2) days after Visit 1. Eligibility criteria were verified again, and CST was repeated for repeatability.

### Exhaled carbon monoxide measurement

The smoking status was objectively verified by measuring exhaled carbon monoxide (eCO) levels (eCO > 7 ppm indicating smoking status) with a portable CO monitor (Micro CO; Micro Medical Ltd, UK). Subjects were asked not to smoke cigarettes for at least 2 h before eCO measurements. Subjects were invited to exhale slowly into a disposable mouthpiece attached to the eCO monitor per the manufacturer’s recommendations. The value of eCO readings was noted.

### Chester Step Test

The Chester Step Test (CST) is a submaximal exercise test that estimates maximal aerobic capacity (maximal oxygen consumption, *V̇*O_2max_) [9, 24]. Before the test, participants’ blood pressure (BP) and resting heart rate (HR) were measured by a junior doctor. Participants were instructed to refrain from eating, smoking, or consuming tea, coffee, or alcohol for at least 2 h prior. Additionally, they were advised to avoid exercise for 24 h to ensure a consistent baseline. Our protocol included a brief, moderate-intensity pre-test warm-up. Participants were then instructed to step on and off a 25 cm gym step in time with the beat of a metronome. They commenced stepping to the metronome beat initially set at 15 beats/minute for 2 min following which heart rate and rating of were recorded. Every 2 min, the stepping rate was gradually increased by 5 steps/minute (from 15 to 35 bpm, a total of 5 stages) to increase HR. HR and rating and perceived exertion (RPE) were recorded at each stage. Providing the participants showed no clear signs of distress the test continues in a progressive manner until the participant reaches a specific HR (80% HR max) or a moderately vigorous level of exertion (RPE below 14). The maximum test duration was 10 min. Aerobic capacity (*V̇*O_2max_) was predicted using the points at or below 80% of the age-estimated HRmax and employing the ‘line of best fit’ linear graph extrapolation technique through the heart rates recorded at the end of each completed stage and the age-estimated HRmax. We used a publicly available Chester Step Test calculator to determine *V̇*O_2max_: https://www.brianmac.co.uk/chester.htm.

### Statistical analysis

This proof-of-concept study was conducted to validate the repeatability of *V̇*O_2max_ using the Chester Step Test (CST) and to increase researchers’ confidence in the value of this test for clinical research, including several clinical trials conducted with smokers at our center of excellence [25, 26]. No previous data for *V*O_2max_ values obtained by CST were available for power calculation. However, based on previous research using bicycle ergometers [20], the extent of changes after quitting smoking was detectable even with a relatively small sample size.

The distribution of the data was assessed by the Kolmogorov–Smirnov test. Counts and percentages summarized gender data; continuously distributed data, with symmetrical distribution, were summarized using the mean (standard deviation; SD); continuously distributed data, with skewed distribution, was summarized using the median (interquartile range; IQR). Clinical data comparisons among study groups were carried out by the Chi-square test for categorical data, one-way ANOVA for continuously symmetric data (age and BMI data), and Kruskal–Wallis test for continuously skewed data (n. cigarette/day, Year smoking, and Pack/Years data). Linear regression analysis was performed between the measurements obtained at V1 and those obtained at V2 to assess the repeatability of *V*O_2max_ in each study group. Scatter plots of linear regression analyses were generated to visualize repeatability results. Moreover, “Bland and Altman” plots were generated to describe the level of agreement between two measurements (V1 and V2). Assessment of the difference from zero of the mean difference between two measurements was performed by a 1-sample *t* test. Pearson’s correlation test was used to evaluate the relationship between body mass index (BMI) and *V*O_2max_ values. Finally, to evaluate the agreement in the repeatability of the intrasession measurements among the two visits (V1 and V2), the intra-class correlation coefficient (ICC) was computed using a single-measurement, absolute-agreement, two-way mixed-effects model [27]. Comparison of *V*O_2max_ among study groups was performed using ANCOVA, adjusting for age and gender, and followed by Tukey’s post hoc comparison test. All analyses were considered significant with a *P *value < 0.05. R version 4.2.3 (2023-03-15) was utilized for data analysis and generation of graphs.

## Results

### Study participants

We enrolled a total of 75 subjects: five (two smokers, two former smokers, and one never-smoker) were not able to complete the minimum requirement of three stages needed to estimate *V*O_2max_. Therefore, the complete analysis of *V*O_2max_ assessment was carried out in 70 subjects (32 females; mean ± SD age of 33.51 ± 7.89 years), including 23 smokers, 23 ex-smokers, and 24 never-smokers (Table 1).

**Table 1:** Clinical characteristics of study groups

	Smokers	Former smokers	Never smokers	*p* value
Subjects n	23	23	24	
Age (yr)	33.48 ± 9.2	32 ± 8.5	35 ± 5.6	0.668
Female	8/23 (34.8%)	12/23 (52.2%)	12/24 (50%)	0.434
N. cigarette/day	15 (10–15)	15 (12–20)	//	0.470
Year smoking	12 (10–22)	10 (5.5–17)	//	0.148
Pack/years	10 (7.5–12.5)	9 (3.75–15)	//	0.443
Year non-smoking	//	2 (1.5–5)	//	NA
Body mass index	28.18 ± 3.5	29.05 ± 3.3	27.97 ± 2.7	0.445

Data are presented as mean ± standard deviation (SD), median (interquartile range), and n/N (%), unless otherwise stated

### ***Repeatability of VO***_***2max***_

**Fig. 1:**
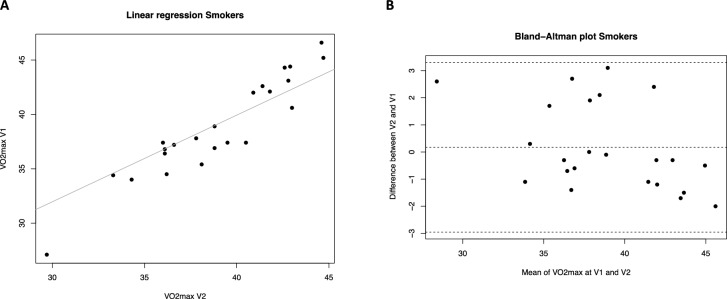
*V*O_2max_ repeatability (V2 *vs* V1) in Smokers. **A** Scatter plot of regression analysis between visit 2 (V2) and visit 1 (V1). **B** Bland Altman plot shows the differences in *V*O_2max_ measurements between V2 and V1 against their mean values for smokers. The average difference is 0.174. The limits of the agreement range from − 2.96 to 3.30

**Fig. 2:**
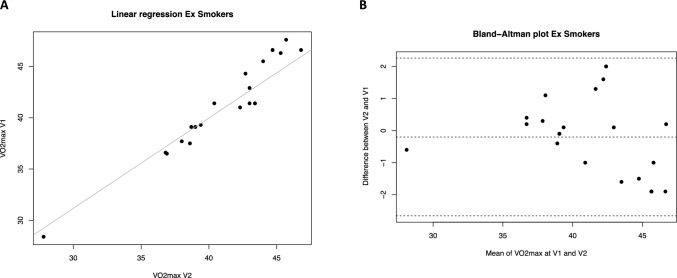
*V*O_2max_ repeatability (V2 *vs* V1) in Ex-smokers. **A **Scatter plot of regression analysis between visit 2 (V2) and visit 1 (V1). **B** Bland Altman plot shows the differences in *V*O_2max_ measurements between V2 and V1 against their mean values for ex-smokers. The average difference is − 0.2. The limits of the agreement range from -2.66 to 2.26

**Fig. 3:**
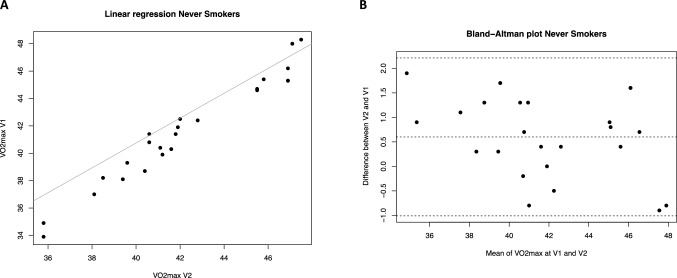
*V*O_2max_ repeatability (V2 *vs* V1) in never-smokers. **A** Scatter plot of regression analysis between visit 2 (V2) and visit 1 (V1). **B** Bland Altman plot shows the differences in *V*O_2max_ measurements between V2 and V1 against their mean values for ex-smokers. The average difference is 0.6. The limits of the agreement range from − 1.01 to 2.21

The repeatability analysis of the *V*O_2max_ parameter was conducted using linear regression models, Bland Altman graphical evaluation, and calculation of the ICC coefficient for the three study groups: smokers, ex-smokers, and never-smokers (Table 2). The linear regression results revealed a strong positive relationship between *V*O_2max_ measurements at visit 1 and visit 2 in all groups (Fig. 1A, 2A and 3A), with high *R*^2^ values indicating a good fit.

**Table 2:** VO_2max_ repeatability

Groups	Repeatability		
	Regression analysis V2-V1 *R* value (*p* value)	Mean of the difference V2-V1 being different from zero? Yes/no (*p* value)	ICC (*p* value)
Smokers	*R* = 0.885 (*p* < 0.0001)	No (*p* = 0.607)	0.930 (*p* < 0.0001)
Former smokers	*R* = 0.928 (*p* < 0.0001)	No (*p* = 0.453)	0.960 (*p* < 0.0001)
Never smokers	*R* = 0.957 (*p* < 0.0001)	Yes (*p* = 0.002)	0.964 (*p* < 0.0001)

For smokers, the Altman-Bland plot revealed an average difference of 0.174 between the two measurements, indicating a slight systematic bias towards higher measurements at visit 2. Nonetheless, the mean difference is not significantly different from zero, suggesting no substantial systematic measurement bias overall. The limits of agreement are [− 2.96, 3.30], indicating that 95% of the differences fall within this range. The differences were randomly distributed around the mean, showing no clear pattern of increasing mean measurements, and no outliers exceeded the limits of agreement (Fig. 1B).

For former smokers, the Altman-Bland plot showed a mean difference of − 0.2, indicating a slight bias towards visit 1 measurements. However, this mean difference is not significantly different from zero, indicating no significant systematic measurement bias. The limits of agreement were [− 2.66, 2.26], with 95% of the differences falling within this range. The differences appeared randomly distributed around the mean, with no obvious pattern of increase or decrease with the mean measurements (Fig. 2B).

For never-smokers, the Altman-Bland plot displayed an average difference of 0.6, indicating a systematic bias toward measurements at visit 2. This mean difference is significantly different from zero (*p* = 0.002), suggesting a consistent variation between the measurements at visit 1 and visit 2. The limits of agreement were [− 1.01, 2.21], with 95% of the differences falling within this range. The distribution of differences was random around the mean, without any obvious pattern related to increasing mean measurements (Fig. 3B). Despite these observations, the high ICC in all groups underscores the overall excellent repeatability and reliability of the *V*O_2max_ measurements.

### ***Correlation between BMI and VO***_***2max***_

The correlation between BMI and *V*O_2max_ was evaluated at the first and second-time points (*V*O_2max_ V1 and V2). The results indicated a weak, non-significant negative correlation (*r* = − 0.064, *p* = 0.601). Similarly, the correlation between BMI and *V*O_2max_ at the second time point (VO_2max_.V2) also showed a weak, non-significant negative correlation (*r* = − 0.112, *p* = 0.354).

It is important to note that the five patients who were excluded from the analysis due to failure to complete the CST had very high BMI, with a mean ± SD of 35.5 ± 2. This exclusion may have influenced the correlation results by reducing the sample size, potentially leading to an underestimation of the true relationship between BMI and *V*O_2max_. Patients with higher BMI might have shown a stronger negative correlation, and their exclusion could introduce selection bias.

### ***Comparison of VO***_***2max***_*** among smokers, ex-smokers and never-smokers***

**Fig. 4:**
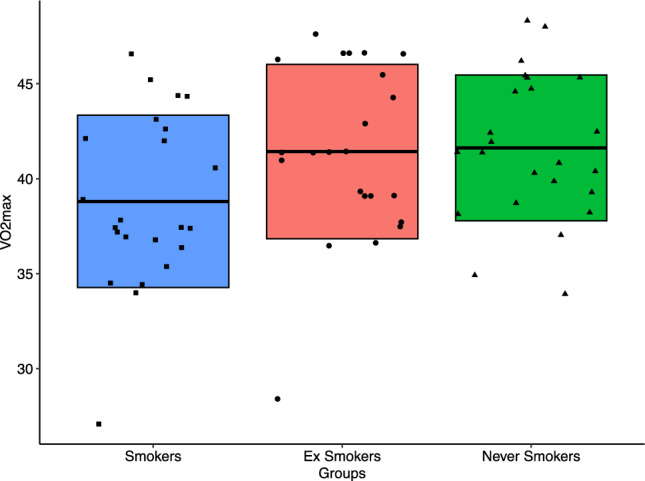
Comparison of *V*O_2max_ among Smokers, Ex-smokers, and Never smokers. Individual *V*O_2max_ values were represented for smokers (■), ex-smokers (●), and never smokers (▲). Boxplots represent the standard deviation above and below the mean value of *V*O_2max_ for each study group

A notable difference in *V*O_2max_ was found among the study groups (*p* < 0.0001) (Fig. 4). Smokers had a mean *V*O_2max_ ± SD of 38.8 ± 4.5, which was significantly lower than the *V*O_2max_ of 41.62 ± 3.8 in never-smokers (*p* < 0.0001) and 41.43 ± 4.6 in ex-smokers (*p* < 0.0001). There was no significant difference in *V*O_2max_ between ex-smokers and never-smokers (*p* = 0.930).

## Discussion

Avoiding cigarette smoke toxicants can lead to measurable improvements in physical performance. This study is the first to investigate the impact of smoking and smoking abstinence on aerobic capacity (*V*O_2max_) using the Chester Step Test. The findings showed that the aerobic capacity of current smokers is significantly lower compared to non-smokers and former smokers.

The observation that the *V*O_2max_ of current smokers is substantially inferior compared to never smokers was not unexpected. This conclusion is consistent with the well-documented compromised oxygen transport caused by high carbon monoxide (CO) levels generated by the combustion process of tobacco cigarettes. Our clinical findings are compatible with previous research using other methods, such as a graded maximal exercise stress test performed on a bicycle ergometer (20) or a treadmill (21, 22) to calculate exercise performance.

Compared to current smokers, both former and never smokers had higher *V*O_2max_ values (indicating better aerobic capacity), with no statistically significant difference between the two groups. Therefore, the inferior aerobic capacity caused by smoking may be reversible, with persistent improvement after stopping smoking. A significant difference between current and former smokers was observed despite the relatively short duration of smoking abstinence (2 years on average) in our sample of former smokers, suggesting that subjects who stop smoking can experience an early beneficial improvement in their aerobic capacity.

The reported difference in *V*O_2max_ between current smokers and former smokers was not only statistically significant but also of practical relevance because it exceeded the 2.0 mL·kg − 1·min − 1 threshold, above which the change in aerobic capacity is considered to be greater than the minimum clinically important difference—MCDI [28]. An improvement beyond the MCID indicates enhanced aerobic capacity, potentially leading to better daily functioning and physical performance.

To our knowledge, no other study has compared *V*O_2max_ among smokers, non-smokers, and former smokers using the CST approach. A recent systematic review has examined exercise capacity in relation to smoking habits using cardiopulmonary exercise testing (CPET) in healthy smokers and found that half of the included studies reported a significant difference in *V*O_2max_ between smokers and non-smokers, with smokers showing lower values, ranging from 1 to 6 mL/kg/min depending on smoking history [29]. The remaining studies showed either no significant differences or inconsistent results.

The use of simpler, reliable tests like the one used in our study offers a scalable approach to exploring these relationships. Clinically, *V*O_2max_ is a strong predictor of exercise capacity, and low *V*O_2max_ is indicative of poor cardiorespiratory function, making our findings highly relevant for health and daily physical activity. Previous research has demonstrated that exercise capacity is a more powerful predictor of mortality in men than other cardiovascular risk factors, and a low *V*O_2max_ is associated with an increased risk of all-cause mortality [30, 31]. For example, studies have shown that even small improvements in physical fitness in healthy middle-aged men significantly reduce the risk of death over 10 years [32]. These findings were recently confirmed in a cohort of 266,109 participants, where a 1 mL·kg − 1·min − 1 increase in *V*O_2max_ reduced the risk of all-cause mortality by 2.3% and cardiovascular morbidity by 2.6% over a 10 year period (33).

This study also investigated the repeatability of *V*O_2max_ values obtained by the Chester Step Test (CST) in current, former, and never smokers. Notably, a high level of *V*O_2max_ repeatability was reported between study visits across all groups. However, in never-smokers, the Bland–Altman plot showed a systematic bias with subjects consistently performing better at V2, suggesting the possibility of a learning effect in this population due to increased familiarity with the step test. The issue of test variability is significant when investigating exercise performance in clinical trials, including those involving smokers. We believe that the good repeatability in this study was due to (1) optimization of testing conditions (ambient temperature, humidity, and time of day were all kept constant); (2) careful consideration of factors that could significantly affect study measurements (e.g., asking participants not to engage in vigorous exercise/sports before and between sessions, and to avoid heavy meals before visits); (3) well-trained subjects performing the step test correctly and consistently; and (4) using a digital pulse oximeter with a soft finger sensor for accurate heart rate monitoring.

A few factors and limitations need to be considered when interpreting these study findings. First, the study populations consisted of relatively young subjects, and their *V*O_2max_ measurements may not be representative of the general population. This is particularly important, considering that age is an important factor influencing *V*O_2max_. Consequently, additional studies with more representative age groups are needed to confirm our findings. Second, due to its cross-sectional design and observational nature, the study results should be interpreted with caution. Nonetheless, our finding that *V̇*O_2max_ values are substantially higher in never and former smokers compared to current smokers is biologically plausible, given the well-known notion that smoking can impair the efficiency of oxygen transportation and utilization during exercise. Third, baseline fitness levels can significantly affect subjects’ performance on a step test, with fitter individuals generally performing better. In this study, a formal assessment of physical activity readiness was not conducted. However, only 5 out of 75 subjects were unable to complete the minimum three stages required to estimate *V*O_2max_ with the CST, and this was primarily due to their high BMI, which averaged 35.5. Fourth, the COVID-19 pandemic had no impact on the study’s conduct because it was paused after ERB approval and launched early in 2022 when most restrictions to hospital access for not-life-saving clinical trials were lifted. Fifth, *V*O_2max_ measurements in former smokers were no different from those of never-smokers, but significant differences could have been detected with a much larger sample size.

## Conclusion

*V*O_2max_ estimates by the Chester Step Test (CST) were reproducible and demonstrated that the aerobic capacity of current smokers is significantly lower compared to never-smokers. The observed improvement in cardiorespiratory performance following smoking cessation suggests that *V*O_2max_ measurements could serve as a valuable biomarker of physiological changes, particularly for smokers who perceive enhanced physical performance as a tangible benefit. Recognizing these changes as early, clinically significant, and reproducible health effect indicators will boost confidence in their value for various clinical, regulatory, and research applications. In particular, early changes in *V*O_2max_ measurements can be investigated in large prospective studies of former smokers abstaining from tobacco cigarettes or switching to combustion-free nicotine products, such as e-cigarettes (ECs) and heated tobacco products (HTPs).

## Data Availability

The de-identified datasets from the trial were sourced from the open science repository maintained by the Center of Excellence for the Acceleration of Harm Reduction (CoEHAR) at the University of Catania, and subsequently utilized for this analysis: https://zenodo.org/records/7941030.
